# MicroRNA-506-3p inhibits osteosarcoma cell proliferation and metastasis by suppressing RAB3D expression

**DOI:** 10.18632/aging.101468

**Published:** 2018-06-10

**Authors:** Wang Jiashi, Qiu Chuang, Zhang Zhenjun, Wang Guangbin, Li Bin, He Ming

**Affiliations:** 1 Department of Orthopedic Surgery, Shengjing Hospital of China Medical University, Shenyang 110004, People’s Republic of China

**Keywords:** RAB3D, osteosarcoma, miR-506-3p, proliferation, metastasis

## Abstract

Osteosarcoma is an aggressive bone tumor primarily affecting children and adolescents. Its cause is not yet fully understood, and there is an urgent need for more effective treatment. In the present study we identified several miRNAs whose expression is altered in osteosarcoma compared to adjacent normal tissue. Moreover, expression levels of one of those miRNAs, miR-506-3p, correlated negatively with expression of RAB3D (a Ras-related protein). Suppression of miR-506-3p in osteosarcoma led to increased expression of RAB3D, which in turn led to increased CDK4 (cyclin-dependent kinase 4) and MMP9 (matrix metalloprotein 9) activities. Our results suggest that miR-506-3p acts as a tumor suppressor in osteosarcoma and that its downregulation leads to tumor cell proliferation and metastasis due to upregulation of RAB3D- and CDK4-mediated signaling. miR-506-3p thus appears be a potentially useful target for adjuvant therapy in osteosarcoma patients.

## Introduction

Osteosarcoma is a highly malignant cancer occurring primarily in adolescents. About 75% of the patients are 15-25 years old, and most are male. The prognosis of osteosarcoma patients is very poor [1], and new and more effective methods of treatment of this disease are greatly needed.

Studies have shown that alterations in miRNA expression are associated with a variety of human diseases, including cancer and nervous and digestive system diseases [2]. In the context of cancer, miR-506 overexpression in HCT116-OxR cells enhances oxaliplatin sensitivity by inhibiting MDR1/P-gp expression via down-regulation of the Wnt/β-catenin pathway and thus provides a rationale for the development of miRNA-based strategies to reverse oxaliplatin resistance in colorectal cancer cells [3]. MiR-506 also suppresses neuroblastoma metastasis by targeting ROCK1 [4], and downregulation of miR-506 expression facilitates pancreatic cancer progression [5]. In osteosarcoma, downregulated miR-506 expression promotes osteosarcoma cell growth through JAG1 [6]. Overexpression of miR-506 suppresses proliferation and promotes apoptosis of osteosarcoma cells by targeting astrocyte elevated gene-1 [7] and suppresses Snail2-mediated osteosarcoma invasiveness [8].

RAB GTPases control exocytic and endocytic membrane trafficking such as exosome release. RAB3D is an essential regulator for protein secretion. In addition, within cancer cells RAB3D activates intracellular AKT/GSK3β signaling to induce growth and metastasis [9]. The role of RAB3D in osteosarcoma progression has never been systematically studied. In the present study, therefore, we examined the relation between miR-506-3p and RAB3D in osteosarcoma tissues and several osteosarcoma cell lines. Our findings suggest miR-506-3p functions as a tumor suppressing by targeting RAB3D expression in osteosarcoma cells. These results provide a theoretical foundation of molecular targeted therapy in osteosarcoma.

## RESULTS

### Identification of differentially expressed miRNAs in osteosarcoma

**Figure 1:**
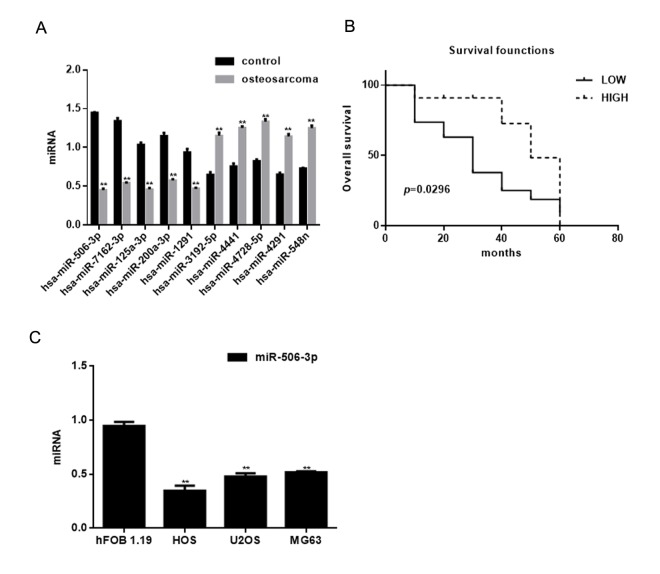
**Identification of miRNAs differentially expressed in osteosarcoma.** (**A**) Expression of miRNAs in osteosarcoma tissues and adjacent normal tissues detected using real time PCR. ** P< 0.05 vs. adjacent tissues group. (**B**) Relationship between miR-506-3p and survival in osteosarcoma patients. (**C**) Expression of miR-506-3p in hFOB 1.19, HOS, U2OS, and MG63 cells detected using real time PCR. ** P< 0.05 vs. hFOB 1.19 cells

The miRNA microarray showed 10 miRNAs to be downregulated (*P*<0.05) and 8 to be overexpressed (*P*<0.05) in osteosarcoma tissues compared with adjacent normal tissues (Table 1). The expression of miRNAs exhibiting >30-fold change between osteosarcoma tissues and adjacent normal tissues was assessed using real time PCR (Figure 1A). Among these, miR-506-3p was decreased significantly in osteosarcoma tissues (Table 2). Moreover, survival analysis showed that patients with higher miR-506-3p expression survived for longer periods (Figure 1B). Consistent with these findings, miR-506-3p expression was also downregulated in osteosarcoma cell lines (Figure 1C).

**Table 1:** Differentially expressed miRNAs in osteosarcomas.

miRNA	*P*	Fold change (osteosarcoma/adjacent tissues)	trend
hsa-miR-506-3p	7.59E-05	83.15854	down
hsa-miR-7162-3p	6.23E-04	63.033234	down
hsa-miR-125a-3p	1.91E-03	53.486415	down
hsa-miR-200a-3p	0.001	52.325701	down
hsa-miR-1291	0.001	44.855346	down
hsa-miR-141-3p	0.013	21.34879	down
hsa-miR-6775-3p	0.022	19.03982	down
hsa-miR-4286	0.023	8.365221	down
hsa-miR-6734-3p	0.025	6.38729	down
hsa-miR-6766-5p	0.042	1.29846	down
hsa-miR-3192-5p	1.98E-06	72.57649	Up
hsa-miR-4441	1.62E-05	60.51947	Up
hsa-miR-4728-5p	1.57E-04	56.24707	Up
hsa-miR-4291	0.001	56.24707	Up
hsa-miR-548n	0.001	41.90057	Up
hsa-miR-5590-5p	0.001	40.66968	Up
hsa-miR-367-5p	0.007	27.48372	Up
hsa-miR-4429	0.029	12.49285	Up

**Table 2:** The relationship between miR-506-3p and osteosarcomas.

Variables	Description	No. of patient	miR-506-3p expression		χ^2^	*P* value
			Low High			
Gender	Male	21	11	10	1.693	0.193
	Female	9	7	2		
Age(years)	<20	18	12	6	0.064	0.800
	≥20	12	6	6		
Family history	No Yes	24 6	14 4	10 2	0.139	0.709
TNM grade	IIA	14	5	9	7.232	0.027*
	IIB III	12 4	9 4	3 0		

### RAB3D is a direct target of miR-627-3p

**Figure 2:**
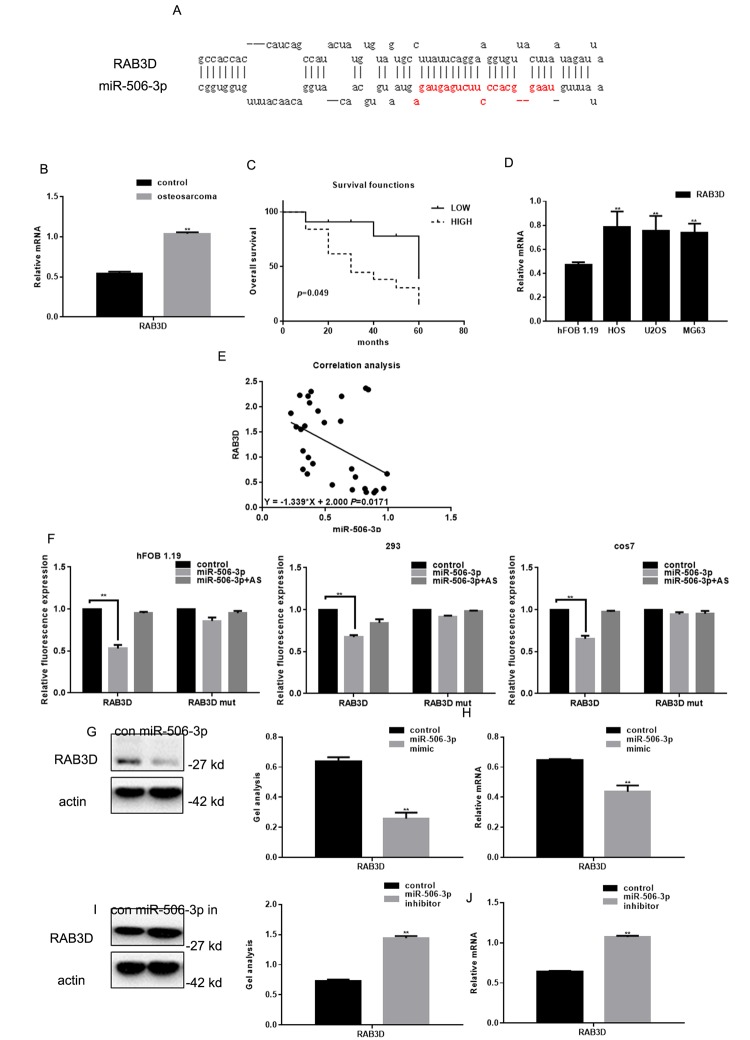
**RAB3D is a direct target of miR-506-3p in osteosarcoma.** (**A**) miRDB predicted miR-506-3p specifically combines with RAB3D. (**B**) Expression of RAB3D mRNA in osteosarcoma and adjacent normal tissues detected using real-time PCR. Data are shown as the mean ± SEM. ** P< 0.05 vs. adjacent tissues. (**C**) Relationship between RAB3D and survival in osteosarcoma patients. (**D**) Expressions of RAB3D in hFOB 1.19, HOS, U2OS, and MG63 cells detected using real time PCR. ** P< 0.05 vs. hFOB 1.19 cells. (**E**) Correlation between expression levels of miR-506-3p and RAB3D in osteosarcoma. (**F**) Interaction between miR-506-3p and the RAB3D 3’-UTR tested in luciferase reporter assays. Data are shown as the mean ± SEM. **P< 0.05 vs. control. (**G-J**) Western blot and real time PCR analyses showing that when miR-506-3p is overexpression/repressed the expression of RAB3D was down/upregulated. Data are shown as mean ± SEM. ** P< 0.05 vs. control.

We found that miR-506-3p could be combined with 3’-UTR of RAB3D using miRDB (Figure 2A). Real time PCR showed that RAB3D expression was significantly higher in osteosarcoma tissues than in adjacent normal tissues (Figure 2B). Survival analysis showed that the patients with lower RAB3D expression survived for longer periods (Figure 1C). We also observed that RAB3D is upregulated in osteosarcoma cell lines (Figure 2D) and that there is a negative correlation between the expression levels miR-506-3p and RAB3D in osteosarcoma (Figure 2E). Luciferase reporter assays showed that miR-506-3p reduces expression of RAB3D at the transcriptional level in hFOB 1.19, HEK 293, and cos7 cells (Fig 2F). When cells were co-transfected with RAB3D and miR-506-3p, luciferase activity was suppressed. However, no suppression was seen when cells were co-transfected with RAB3Dmut or miR-506-3p antisense. In addition, when we transfected miR-506-3p mimics or miR-506-3p inhibitors into osteosarcoma cells, western blotting and real-time PCR showed that RAB3D expression suppressed by miR-506-3p mimics but enhanced by miR-506-3p inhibitors (Figure 2G-J).

### MiR-506-3p inhibits osteosarcoma cell proliferation

**Figure 3:**
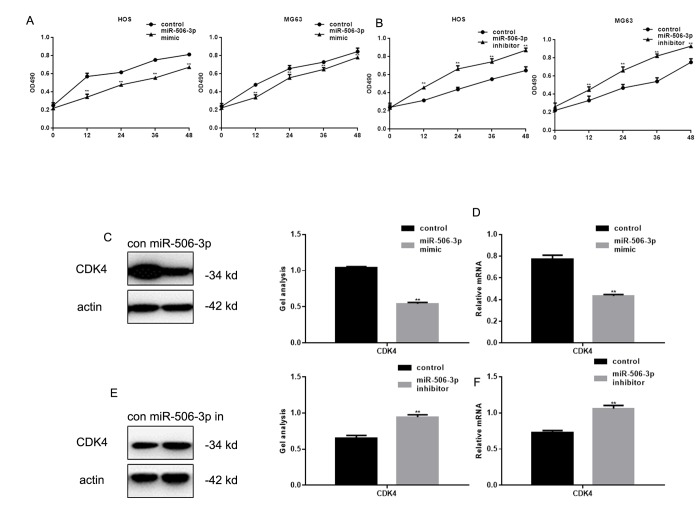
**MiR-506-3p inhibited proliferation of osteosarcoma cells.** (**A, B**) MTT assay showing effect of transfecting miR-506-3p mimic/inhibitor on HOS and MG63 cell proliferation. Data are shown as the mean ± SEM. ** P< 0.05 vs. control. (**C-F**) Western blot and real-time PCR analyses showing the effect of osteosarcoma cell transfection with miR-506-3p mimic/inhibitor expression of CDK4. Data are shown as mean ± SEM. ** P< 0.05

MTT assays showed that transfection of miR-506-3p mimics inhibited HOS and MG63 cell proliferation (Figure 3A, B). Previous studies showed that RAB3D stimulates cell cycle progression through effects on CDK4 signaling [11]. Consistent with that finding, we observed that miR-506-3p suppressed CDK4 expression in HOS cells (Figure 3C-F).

### MiR-506-3p inhibits osteosarcoma metastasis

**Figure 4:**
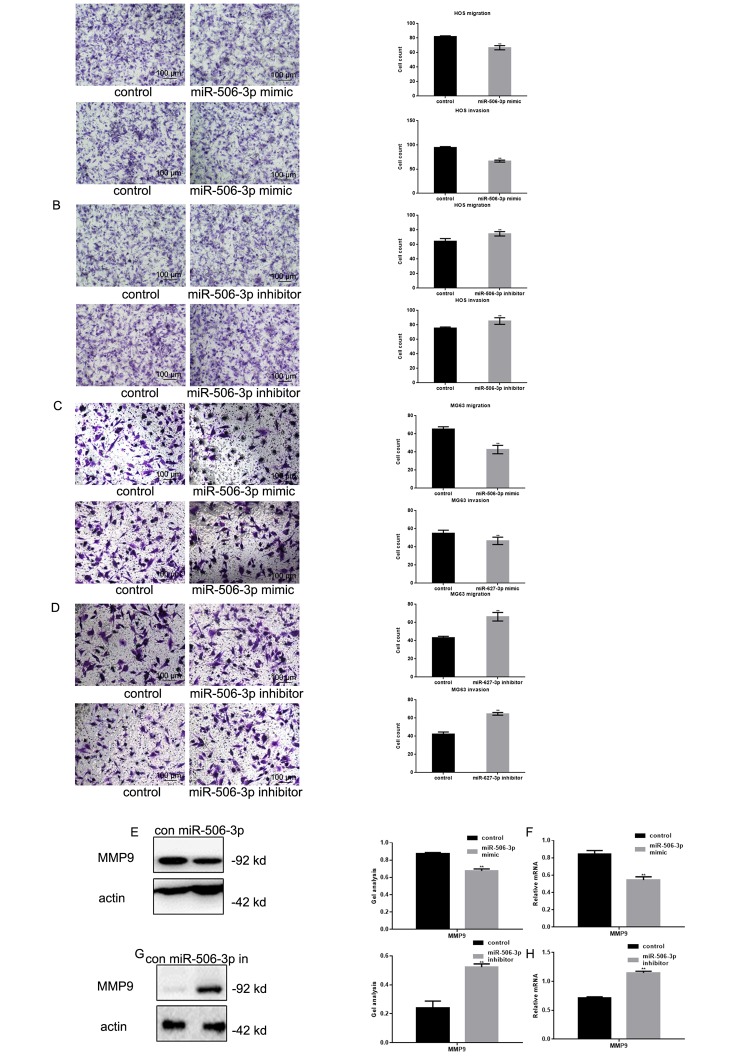
**MiR-506-3p inhibited metastasis of osteosarcoma cells.** (**A-D**) Transwell assays with and without Matrigel showing the effect of miR-506-3p expression/repression on the migration and invasiveness of HOS and MG63 cells. Cells were counted and results are the mean ± SD of three experiments. ** P< 0.05. (**E-H**) Western blot and real-time PCR analysis of MMP2 expression in osteosarcoma cells transfected with miR-506-3p mimic/inhibitor. Data are shown as the mean ± SEM. ** P< 0.05.

Transwell assays showed that transfecting HOS and MG63 cells with miR-506-3p mimics suppressed their Matrigel invasion as compared to transfection with scrambled controls (Figure 4A-D). Conversely, the miR-506-3p inhibitor significantly increased the invasiveness of HOS and MG63 cells. Western blot and real-time PCR showed that MMP9 was significantly downregulated by miR-506-3p (Figure 4E-H).

### Effects of miR-627-3p siRNA and PTN siRNA on the proliferation and metastasis of HOS cells

**Figure 5:**
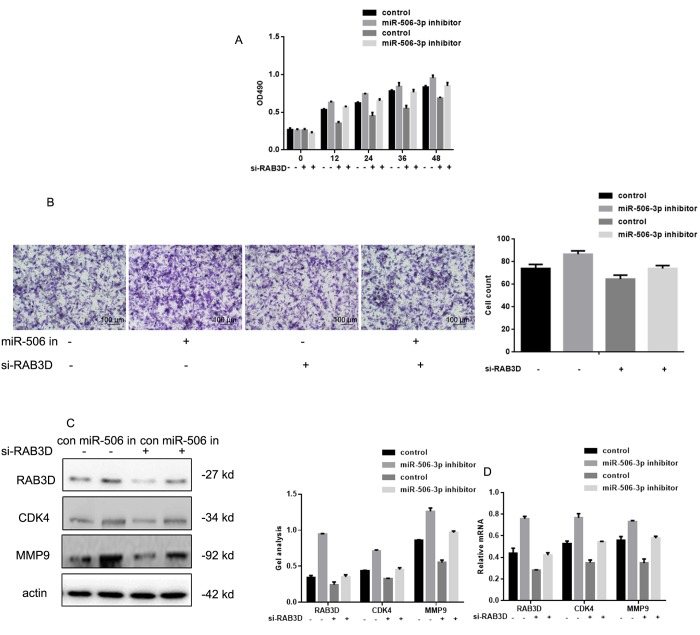
**Effects of miR-506-3p and RAB3D siRNAs on HOS cell proliferation and metastasis.** (**A**) MTT assays showing the effects of miR-506-3p and RAB3D siRNAs on cell proliferation. Data are shown as the mean ± SEM. (**B**) Transwell assays showing the effects of miR-506-3p and RAB3D siRNAs on cell invasiveness. Cells were counted and results represent the mean ± SD of three experiments. (**C, D**) Western blot and real-time PCR analyses showing the effects of miR-506-3p and RAB3D siRNAs on RAB3D, CDK4 and MMP2 were detected by. Data are shown as mean ± SEM.

After co-expressing miR-506-3p inhibitors/control and si-RAB3D/control in HOS cells, MTT and transwell experiments showed that silencing RAB3D suppressed HOS cell proliferation and invasiveness (Figure 5A, B). When RAB3D and miR-506-3p were inhibited simultaneously, cells proliferation and invasiveness was somewhat higher. Western blotting and real time PCR showed that miR-506-3p expression had similar suppressive effects on RAB3D, CDK4 and MMP2 in HOS cells (Figure 5C, D).

### MiR-627-3p inhibits HOS cell invasion in vivo

**Figure 6:**
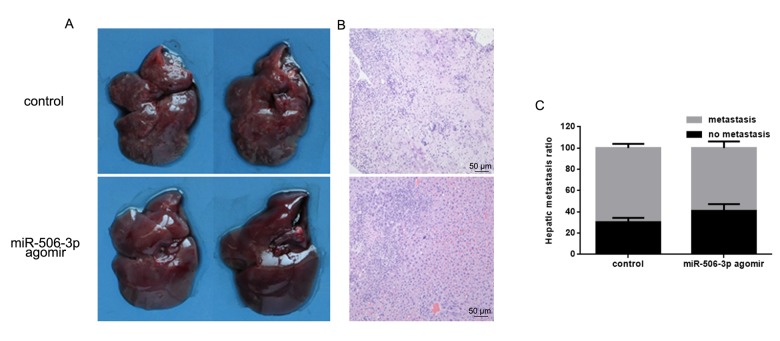
**MiR-506-3p inhibits HOS cell invasiveness in vivo.** (**A, B**). Livers were dissected and macroscopically photographed or stained with hematoxylin and eosin. (**C**) Statistical results for the number of metastasis.

To examine the effect of miR-506-3p on osteosarcoma cell invasiveness in vivo, HOS cells stably expressing vector (control) or miR-627 agomir were injected into nude mice via the tail vein. Histological analysis of liver tissues were collected 21 days later showed lower levels of hepatic invasion by HOS cells in mice injected with miR-506-3p agomir cells (Figure 6A). This finding was confirmed by hematoxylin and eosin staining which showed that as compared to control, miR-506-3p suppressed metastatic spread of HOS cells to the livers (Figure 6B, C).

## DISCUSSION

The development and progression of osteosarcoma appears to be affected by a variety of factors, including miRNAs, which are known suppress/stimulate cell proliferation and/or metastasis of several cancers. In the present study, our microarray analysis showed that miR-506-3p expression is frequently suppressed in human osteosarcoma and that there is a negative correlation between miR-506-3p expression and survival among osteosarcoma patients.

miR-506-3p reportedly suppresses the development and progression of tumors by targeting such proteins as LAMC1 and COLT1 [12,13]. Previously studies observed that RAB3D plays a key role in osteosarcoma [14,15]. To extend that observation, we assessed the relation between miR-506 and RAB3D, a member of the Rab GTPase family, which regulate membrane trafficking. Using miR-506-3p gain- and loss-of-function approaches as well as a tumor model in nude mice, we revealed that miR-506-3p suppresses the proliferation and invasiveness of osteosarcoma cells by targeting RAB3D transcription. Silencing of RAB3D suppresses the proliferation and invasiveness of esophageal squamous cell carcinoma cells [16]. Conversely, high RAB3D expression is associated with tumor progression and is predictive of a poor prognosis in colorectal cancer [11]. Moreover, RAB3D levels are much higher in breast, prostate, lung, colon, ovary, liver and skin carcinoma tissues than in corresponding normal tissues [17–19]. This suggests RAB3D could potentially serve as a broad-spectrum diagnostic biomarker for tumor progression relevant to both patient diagnosis and treatment [9]. Studies have showed that RAB3D influences cell proliferation by stimulating cell cycle progression through effects on CDK4 and CDK6 signaling [20,21]. In addition, RAB3D appears to promote invasion by enhancing MMP expression [22,23].

In summary, our results suggest that miR-506-3p acts as a tumor suppressor in osteosarcoma and that its downregulation leads to tumor cell proliferation and metastasis due to upregulation of RAB3D- and CDK-mediated signaling. miR-506-3p thus appears be a potentially useful target for adjuvant therapy in osteosarcoma patients.

## MATERIALS AND METHODS

### Tissue samples and cell lines

Primary osteosarcoma tumor samples and adjacent normal tissues were obtained from 30 patients in the Shengjing Hospital. The average age of the patients was 21.21 years (range: 11 to 41 years), and 21 of the patients were male. More than 90% of osteosarcomas occurred in extremities. All tumor samples were high-grade osteosarcomas at stage IIA or IIB in the Enneking system, except four tumors were stage III. The osteosarcoma subtypes included conventional (43.33%, 13/30), osteoblastoma-like (26.67%, 8/30), chondroblastoma-like (23.33%, 7/30), fibroblastic (3.33%, 1/30), and fibrohistiocytic (3.33%, 1/30). This study was approved by the Research Ethics Committee of Shengjing Hospital, and all patients provided written informed consent.

hFOB 1.19, 293, cos7, HOS, U2OS and MG63 cells were purchased from the cell bank of the Chinese Academy of Sciences. Cells were incubated at 37°C under a 5% CO_2_ atmosphere and passaged every 2-3 days.

### RNA isolation

Total RNAs including miRNAs were isolated from frozen tissues using Trizol according to the manufacture's protocol. Samples were then stored at -80°C until use.

### MicroRNA microarray analysis

miRNA microarray analysis was outsourced to RiBio Cor (Ribobio, China). Briefly, total RNA extract was purified from the tissues using a mirVana™ miRNA Isolation Kit (TIANGEN, China) and then labeled and hybridized using an Agilent miRNA Complete Labeling and Hybridization Kit (Agilent Technologies, USA). The labeled RNA was examined using an Axon GenePix 4000B microarray scanner. miRNAs whose expression levels changed at least 2-fold between the tumor specimens collected before and after chemotherapy for used for further analysis [24].

### Real-time PCR

To detect expression of miRNAs and mRNAs, real time PCR was performed using a Reverse Transcription Kit Ribobio (Ribobio, China) according to the manufacturer's instructions. After treating samples with DNase I, cDNA was reverse transcribed from the total RNA using a miRNA-specific primer with a TaqMan® microRNA reverse transcription kit (Ribobio, China). The real-time PCR reaction mixture (10 μl) contained 0.5 μl of RT product, 5 μl of TaqMan 2× Universal PCR Master Mix (Ribobio, China), and 1 μl of TaqMan miRNA Assay reagent (Ribobio, China). Real-time PCR protocol entail incubation at 95°C for 10 min, followed by 50 cycles at 95°C for 15 s and 60°C for 1 min. U6 and β-actin were used as internal controls. Expression of miRNAs was detected using a Stem-Loop RT-PCR assay as described previously [25,26]. Primer sequences are listed in Table 3.

**Table 3:** Primer sequences.

Name	Forward primer(5'->3')	Reverse primer(5'->3')
hsa-miR-506-3p	ACACTCATAAGGCACCCTTC	TCTACTCAGAAGGGGAGTAC
hsa-miR-7162-3p	CACTCATCTGAGGTGGAAC	GCTGCTGTTCCGAGTACAT
hsa-miR-125a-3p	ACTCACAGGTGAGGTTC	GGCTGGGAAGGAGAGTAC
hsa-miR-200a-3p	CACTCTAACACTGTCTGG	ACATCGTTACCGAGAGTA
hsa-miR-1291	CACTCTGGCCCTGACTG	ACTGCTGGTCTTGAGTACA
hsa-miR-3192-5p	ACACTCTCTGGGAGGTT	TTCCACTGCTAGAGTACAT
hsa-miR-4441	ACACTCTACAGGGAGGAG	TACAATCTCCGAGAGTAC
hsa-miR-4728-5p	ACACTCTGGGAGGGGAGAG	TGCTTGCTGGAGAGTACA
hsa-miR-4291	ACTCTTCAGCAGGAACAG	AGCTGTTCCTGGAGAGTAC
hsa-miR-548n	ACTCCAAAAGTAATTGTGG	ACAAAATCCACGAGAGTAC
U6	CTCGCTTCGGCAGCACA	ACGCTTCACGAATTTGC
RAB3D	GACCTCCGGTTTAGAGGCAC	GTTGGTTGGTGTTTGGGAGC
CDK4	TGATGCGCCAGTTTCTAAGAGG	GGTCGGCTTCAGAGTTTC
MMP9	CCTGGAGACCTGAGAACCAATC	CCACCCGAGTGTAACCATAGC
β-actin	CATCCTGGCCTCGCTGT	GCTGTCACCTTCACCGTTCC

All the reactions were carried out as described previously [27].

### Western blot analysis

Treated cells were collected by centrifugation at 300× g for 10 min and then lysed using radio immunoprecipitation assay buffer (Beyotime, China) containing 2 mM phenylmethanesulfonyl fluoride. Proteins in samples of lysate were resolved by 10% SDS-PAGE and transferred onto PVDF membranes. After incubation in blocking solution (4% bovine serum albumin in Tris-buffered saline and Tween-20) for 2 h at room temperature, the membranes were incubated with primary antibodies overnight at 4°C. Thereafter, the membranes were incubated with secondary antibodies for 2 h at room temperature. The primary antibodies used were anti-RAB3D (1:500), anti-CDK4 (1:500), anti-MMP9 (1:500), and anti-β-actin (1:2000). HRP-conjugated anti-mouse (1:5000) and anti-rabbit (1:5000) served as secondary antibodies (Santa Cruz, USA). Expression levels of proteins were quantified and normalized to levels of β-actin.

### Dual luciferase reporter assay

Dual luciferase activity assays were performed as described previously [27]. The RAB3D 3’-UTR was PCR amplified and cloned into the pMIR-REPORTTM vector (Ambion, China). The primers used were as follows: for RAB3D-wt, 5’- TGGTGGGGAACAAGTGTGAC -3’ (forward) and 5’- GGAATGAGCCATGCAGGAGT -3’ (reverse); for RAB3D-mut (the miR-506-3p binding site was mutated), 5’- CCCCAAGATCCACATCACCC -3’ (forward) and 5’- CCCTGCCGATGACAAGGATT -3’ (reverse). Twenty-four hours before transfection, 1×10^4^ MG63 cells were plated in a 96-well plate. miR-506-3p mimics, inhibitors or control was transfected into cells together with 100 ng of RAB3D-wt or RAB3D-mut. Luciferase activity was assessed using a Dual Luciferase Reporter Assay System (Promega, USA) 24 h after transfection.

### MTT assays

After transfecting HOS or MG63 cells with miR-506-3p mimics or inhibitors, the cells were seeded onto 96-well plates to a density of 2×10^3^ cells per well and incubated in the corresponding medium supplemented with 10% FBS. After incubation for 0, 12, 24 or 48 h, 10 µl of MMT was adding into each well, and the cells were incubated for an additional 4 h. Absorbance at 490 nm was then measured. Experiments were carried out in triplicate.

### Transwell assay

HOS and MG63 cells were transfected with miRNA mimics, inhibitors or control. Twenty-four hours after transfection, 1×10^5^ cells in serum-free medium were seeded into transwell chambers with or without a Matrigel-covered membrane. After 8 h, the cells were fixed with 4% paraformaldehyde, stained with 0.4% trypan blue, and counted using a microscope.

### Nude mice experiment

Five- to six-week-old female, athymic nude BALB/c mice (Vital River Laboratory Animal Technology Co. Ltd., China) were used to study tumor metastasis. Mice administered 2×10^6^ cells suspended in 1 ml saline via the tail vein were divided into miR-506-3p agomir and control groups (n=6 in each group). On day 21 after tumor cell injection, liver samples were collected for histological examination.

All experimental procedures involving animals were conducted in accordance with the Guide for the Care and Use of Laboratory Animals (NIH publication no. 80-23, revised 1996) and were performed according to the institutional ethical guidelines for animal experiments.

### Histopathology

Liver specimens were fixed with 4% paraformaldehyde. Serial sections (2 μm) were cut using a microtome and affixed onto positively charged slides. All slides were incubated at 60°C for several hours to allow the sections to adhere to the slides. Tissues sections were deparaffinized and rehydrated through a graded xylene and alcohol series. Hematoxylin-eosin staining was performed using standard protocols.

### Statistical analysis

All statistical analyses were performed using SPSS software, version 17.0 (SPSS Inc., Chicago, IL, USA). Data are presented as the mean ± standard deviation. Two groups were compared using Student's t test (two-tailed) and one-way ANOVA. Multiple comparisons were made using one-way ANOVA and the LSD-t method. Statistical significance was defined as P<0.05. All experiments were repeated three times.

### Ethics approval and consent to participate

Research involving human subjects, human material, or human data, was performed in accordance with the Declaration of Helsinki and was approved by the Research Ethics Committee of Shengjing Hospital (R20160965).

### Availability of data and material

No restriction on data or material availability.

### Consent for publication

Written informed consent for the publication of all manuscript details was obtained from Wang Jiashi, Qiu Chuang, Zhang Zhenjun, Wang Guangbin, Li Bin and He Ming.

## References

[1] Andersen GB, Knudsen A, Hager H, Hansen LL, Tost J. miRNA profiling identifies deregulated miRNAs associated with osteosarcoma development and time to metastasis in two large cohorts. Mol Oncol. 2018; 12:114–31 10.1002/1878-0261.12154 29120535 PMC5748490

[2] Wang M, Xie R, Si H, Shen B. Integrated bioinformatics analysis of miRNA expression in osteosarcoma. Artif Cells Nanomed Biotechnol. 2017; 45:936–43. 10.1080/21691401.2016.1196456 27315542

[3] Zhou H, Lin C, Zhang Y, Zhang X, Zhang C, Zhang P, Xie X, Ren Z. miR-506 enhances the sensitivity of human colorectal cancer cells to oxaliplatin by suppressing MDR1/P-gp expression. Cell Prolif. 2017; 50:e12341. 10.1111/cpr.12341 28217977 PMC6529089

[4] Li D, Cao Y, Li J, Xu J, Liu Q, Sun X. miR-506 suppresses neuroblastoma metastasis by targeting ROCK1. Oncol Lett. 2017; 13:417–22. 10.3892/ol.2016.5442 28123576 PMC5245134

[5] Li J, Wu H, Li W, Yin L, Guo S, Xu X, Ouyang Y, Zhao Z, Liu S, Tian Y, Tian Z, Ju J, Ni B, Wang H. Downregulated miR-506 expression facilitates pancreatic cancer progression and chemoresistance via SPHK1/Akt/NF-κB signaling. Oncogene. 2016; 35:5501–14. 10.1038/onc.2016.90 27065335 PMC5078861

[6] Hu M, Yuan X, Liu Y, Tang S, Miao J, Zhou Q, Chen S. IL-1β-induced NF-κB activation down-regulates miR-506 expression to promotes osteosarcoma cell growth through JAG1. Biomed Pharmacother. 2017; 95:1147–55. 10.1016/j.biopha.2017.08.120 28926924

[7] Yao J, Qin L, Miao S, Wang X, Wu X. Overexpression of miR-506 suppresses proliferation and promotes apoptosis of osteosarcoma cells by targeting astrocyte elevated gene-1. Oncol Lett. 2016; 12:1840–48. 10.3892/ol.2016.4827 27602115 PMC4998420

[8] Yu Z, Zhang Y, Gao N, Wang X. Overexpression of miR-506 inhibits growth of osteosarcoma through Snail2. Am J Transl Res. 2015; 7:2716–23. 26885269 PMC4731669

[9] Yang J, Liu W, Lu X, Fu Y, Li L, Luo Y. High expression of small GTPase Rab3D promotes cancer progression and metastasis. Oncotarget. 2015; 6:11125–38. 10.18632/oncotarget.3575 25823663 PMC4484444

[10] Pfaffl MW. A new mathematical model for relative quantification in real-time RT-PCR. Nucleic Acids Res. 2001; 29:e45. 10.1093/nar/29.9.e45 11328886 PMC55695

[11] Luo Y, Ye GY, Qin SL, Mu YF, Zhang L, Qi Y, Qiu YE, Yu MH, Zhong M. High expression of Rab3D predicts poor prognosis and associates with tumor progression in colorectal cancer. Int J Biochem Cell Biol. 2016; 75:53–62. 10.1016/j.biocel.2016.03.017 27046094

[12] Zu C, Liu T, Zhang G. MicroRNA-506 Inhibits Malignancy of Colorectal Carcinoma Cells by Targeting LAMC1. Ann Clin Lab Sci. 2016; 46:666–74. 27993882

[13] Guo S, Yang P, Jiang X, Li X, Wang Y, Zhang X, Sun B, Zhang Y, Jia Y. Genetic and epigenetic silencing of mircoRNA-506-3p enhances COTL1 oncogene expression to foster non-small lung cancer progression. Oncotarget. 2017; 8:644–57. 10.18632/oncotarget.13501 27893417 PMC5352185

[14] Taylor A, Mules EH, Seabra MC, Helfrich MH, Rogers MJ, Coxon FP. Impaired prenylation of Rab GTPases in the gunmetal mouse causes defects in bone cell function. Small GTPases. 2011; 2:131–42. 10.4161/sgtp.2.3.16488 21776414 PMC3136943

[15] Zhao H, Ettala O, Väänänen HK. Intracellular membrane trafficking pathways in bone-resorbing osteoclasts revealed by cloning and subcellular localization studies of small GTP-binding rab proteins. Biochem Biophys Res Commun. 2002; 293:1060–65. 10.1016/S0006-291X(02)00326-1 12051767

[16] Zhang J, Kong R, Sun L. Silencing of Rab3D suppresses the proliferation and invasion of esophageal squamous cell carcinoma cells. Biomed Pharmacother. 2017; 91:402–07. 10.1016/j.biopha.2017.04.010 28472755

[17] Larkin JM, Woo B, Balan V, Marks DL, Oswald BJ, LaRusso NF, McNiven MA. Rab3D, a small GTP-binding protein implicated in regulated secretion, is associated with the transcytotic pathway in rat hepatocytes. Hepatology. 2000; 32:348–56. 10.1053/jhep.2000.9110 10915742

[18] Luo Y, Yu SY, Chen JJ, Qin J, Qiu YE, Zhong M, Chen M. MiR-27b directly targets Rab3D to inhibit the malignant phenotype in colorectal cancer. Oncotarget. 2017; 9:3830–41. 10.18632/oncotarget.23237 29423086 PMC5790503

[19] Martelli AM, Baldini G, Tabellini G, Koticha D, Bareggi R, Baldini G. Rab3A and Rab3D control the total granule number and the fraction of granules docked at the plasma membrane in PC12 cells. Traffic. 2000; 1:976–86. 11208087

[20] da Silva SD, Marchi FA, Xu B, Bijian K, Alobaid F, Mlynarek A, Rogatto SR, Hier M, Kowalski LP, Alaoui-Jamali MA. Predominant Rab-GTPase amplicons contributing to oral squamous cell carcinoma progression to metastasis. Oncotarget. 2015; 6:21950–63. 10.18632/oncotarget.4277 26110570 PMC4673138

[21] Boulay PL, Mitchell L, Turpin J, Huot-Marchand JE, Lavoie C, Sanguin-Gendreau V, Jones L, Mitra S, Livingstone JM, Campbell S, Hallett M, Mills GB, Park M, et al. Rab11-FIP1C Is a Critical Negative Regulator in ErbB2-Mediated Mammary Tumor Progression. Cancer Res. 2016; 76:2662–74. 10.1158/0008-5472.CAN-15-2782 26933086 PMC5070470

[22] Zhang D, Lu C, Ai H. Rab5a is overexpressed in oral cancer and promotes invasion through ERK/MMP signaling. Mol Med Rep. 2017; 16:4569–76. 10.3892/mmr.2017.7214 28849149 PMC5646994

[23] Wiesner C, El Azzouzi K, Linder S. A specific subset of RabGTPases controls cell surface exposure of MT1-MMP, extracellular matrix degradation and three-dimensional invasion of macrophages. J Cell Sci. 2013; 126:2820–33. 10.1242/jcs.122358 23606746

[24] Meng Y, Gao R, Ma J, Zhao J, Xu E, Wang C, Zhou X. MicroRNA-140-5p regulates osteosarcoma chemoresistance by targeting HMGN5 and autophagy. Sci Rep. 2017; 7:416. 10.1038/s41598-017-00405-3 28341864 PMC5428500

[25] Feng J, Wang K, Liu X, Chen S, Chen J. The quantification of tomato microRNAs response to viral infection by stem-loop real-time RT-PCR. Gene. 2009; 437:14–21. 10.1016/j.gene.2009.01.017 19374024

[26] Chen C, Ridzon DA, Broomer AJ, Zhou Z, Lee DH, Nguyen JT, Barbisin M, Xu NL, Mahuvakar VR, Andersen MR, Lao KQ, Livak KJ, Guegler KJ. Real-time quantification of microRNAs by stem-loop RT-PCR. Nucleic Acids Res. 2005; 33:e179. 10.1093/nar/gni178 16314309 PMC1292995

[27] Yang TS, Yang XH, Wang XD, Wang YL, Zhou B, Song ZS. MiR-214 regulate gastric cancer cell proliferation, migration and invasion by targeting PTEN. Cancer Cell Int. 2013; 13:68. 10.1186/1475-2867-13-68 23834902 PMC3716801

